# Supplementary material for: Direct measurement forest carbon protocol: a commercial system-of-systems to incentivize forest restoration and management
Source: PeerJ. 2020 Apr 27;8:e8891. doi: 10.7717/peerj.8891 (PMC7192159; doi:10.7717/peerj.8891)
Supplement: Supplemental Information 1 — The System of Systems (SoS), the Global Monitoring Platform (GMP), the eddy covariance method and the Project Management Plan are described, amplifying sections in the narrative. [file peerj-08-8891-s001.docx]

#### Supplement 1. Direct Measurement Forest Carbon Protocol: A Commercial System-of-Systems to Incentivize

**Forest Restoration and Management**

System-of-Systems. Figure 1 shows an SoS sensor network as the central feature of the DMFCP. The SoS integrates GMP's deployed across the project landscape and operates according to the DMFCP. The SoS operation is supported by system operators, calibration analysts and mission data analysts responsive to environmental, regulatory and financial scenarios. The GMP functions are carried out and integrated by central reference, command and control and data processing facilities hosted remotely. A data communication and management subsystem (DCMS) controls data content of the SoS. The DCMS gathers real-time data from the GMP’s and related sensors of the SoS, including analytical uncertainties across processes, in a central location where the data are qualified, analyzed and archived. The DCMS provides data to model analysts to calculate absolute carbon sequestration and utilize applicable carbon sequestration models including carbon isotopes (Davidson et al., 2016; Wang et al., 2016) to characterize the spatial and temporal components of carbon sequestration across the project landscape. The DCMS provides secure digital access to data, charts, figures and images resulting from the project reporting on physical, project, financial and registered carbon. The underlying research data files may also be accessible as authorized through the DCMS. The benefits of a comprehensive data management system include harmonization of processes across all sensor nodes, ensuring data integrity, storage and security resulting in reduced risks for all stakeholders.

The Global Monitoring Platform. The GMP of the DMFCP integrates commercial off-the-shelf gas

analyzers (e.g., 12C16O_2_, 16O12C18O, 13C16O_2_, 12CH_4_, 13CH_4_, 14N_2_16O, 15N_2_16O, O_2_ and hydrocarbons (c2-c9)), prototypes for portable and field measurement of 14CO_2_ (Galli et al. 2013; Genoud et al. 2015; McCartt et al. 2016; Fleisher et al. 2017), an eddy covariance system and micro-meteorological station creating modular, stationary and mobile base stations readily deployable upon initiation of a project. CO_2_ gases are typically measured as parts per million (ppm) or expressed as isotope ratios (e.g. 13/12C, 14/12C)iii. The benefits of the GMP analyzer as a component of the SoS include a standardized, low cost instrument platform for the primary greenhouse gases, automated operation and data reporting in real-time with verified analyzer function through shared reference gas standards and third-party verification. The standardized, modular system is deployable across small and large landscapes (e.g., ~100 to 1,000,000+ 710 hectares) reducing barriers to short and long-term deployment for GHG emission reduction management and verification projects. The GMP is connected to a central reference facility, a data processing facility, a command and control facility and to other GMP's in the project network. The GMP typically includes analyzers for methane (CH_4_) and nitrous oxide (N_2_O) in addition to CO_2_ providing a comprehensive GHG budget for projects recognizing that reductions in CO_2_ may be offset by increases in CH_4_ and N_2_O (Niklaus et al., 2016; Tian et al., 2015; Tupek et al., 2015). The flux methodology for CO_2_ applies to CO_2_ isotopologues including oxygen isotopic species not described here (e.g., 18O12C16O) (Wehr et al., 2013) and to GHG’s and their isotopologues (Arata et al., 2016; Wolf et al., 2015). The GMP employs internal and shared standard reference gas modules for the CO_2_ isotopologues providing standards for reference baseline and calibration for the measured amounts of 12C, 13C and 14C isotopic species for each GMP analyzer within the SoS sensor network. In addition, a key technology feature of the DMFCP includes synchronous measurement of third-party reference gases obtained from national and international entities such as the World Meteorological Organization, the National Oceanic and Atmospheric Association and the National Institute of Standards (e.g., "WMO"; "NOAA"; "NIST"). Such reference gases analyzed by the GMP's ensure transparency, accountability and comparability of data and resulting financial products across all GMP networks on local to global scales. The GMP will house 14CO_2_ analyzer field prototypes, as available, for forest carbon projects to provide measurement of the three molecular forms of CO_2_ defining the natural and anthropogenic carbon cycles. Measurements of 14CO_2_ in the atmosphere, soil atmosphere and as organic carbon may also be analyzed by accelerator mass spectrometry (AMS) where applicable when a portable or field analyzer is not available.

Eddy Covariance (EC). The EC method measures gas fluxes in and out of an ecosystem (D. D. Baldocchi, 2010; Burba, 2013; Running et al., 1999). The EC method is the most accurate and direct approach available for determining the dynamic net ecosystem exchange (NEE) for a project area (Figure 1). The method is based on direct and fast measurements (e.g., 10 Hz) of actual gas transport characterized by a three-dimensional wind field in real time. The concentration of the gas of interest (e.g., CO2, 13CO2 and 14CO2) is measured concomitantly resulting in flux of the gas. In the case of isotopic forms of CO2, isoflux for each isotope is determined (e.g., 13CO2, 14CO2) and employed to create unique DMFCP carbon products based on a two carbon species approach (13/12CO2, 14/12CO2). The EC method has been applied worldwide under remote and harsh conditions employing solar power for months without maintenance (Burba, 2013). Open or closed path gas analyzers (e.g., CO2, CH4, N2O) coupled with automated flux calculation, telemetry and integrated micrometeorological sensors, for example, could be employed in DMFCP systems serving as initial base platforms readily delivered to the project site. Additional instrumentation could be integrated as specified in the project plan. EC data are analyzed by a variety of models across small and large-scale to calculate NEE (Burba, 2013; Fox et al., 2009). Off the shelf bulk and isotopic analyzers for EC measurements are available from a variety of vendors (e.g., Licor Inc, Lincoln, NE, USA; Campbell Scientific Campbell Scientific Inc., Logan, UT, USA; Picarro, Santa Clara 746 CA, USA; Los Gatos Research, San Jose, CA, USA). Uncertainty for eddy covariance NEE is well described across all variables involved (<https://fluxnet.fluxdata.org/data/fluxnet2015-dataset/fullset-data-product/>) yielding quality assurance/quality control for single variables and multiple and combined variables (<https://fluxnet.fluxdata.org/data/fluxnet2015-dataset/data-processing/>). Open source code for eddy covariance data processing such as ONEFlux is freely available under a BSD license (e.g., https://github.com/AmeriFlux/ONEFlux). ONEFlux (Open Network-Enabled Flux processing pipeline) automatically consolidates multiple computations to process half-hourly flux inputs including friction-velocity-threshold estimation methods and filtering, gap-filling of micrometeorological and flux variables, partitioning of CO_2_ fluxes into ecosystem respiration and gross primary production and related uncertainties. ONEFlux is compatible with and has been tested against the FLUXNET2015 dataset.

Project Management Plan. A project is initiated with a Memorandum of Understanding and a Project Listing Application describing details of the project location, proof of ownership or management authority, ecology and historical and current forest operations. A Project Management Plan is created that reflects the project agreement and contract terms to reverse deforestation through payments made to landowners in lieu of forest harvest and destruction. Deployment of the SoS with GMP nodes is established. Data quality standards and reporting of data for monetization to carbon markets are established. Terms related to payments to landowners are agreed upon including options for upfront, and upfront plus royalty on sales, including schedule of payments are defined. For example, annual payments with the flexibility to defer payments over two to five-year intervals may apply to forests with intermittent net positive emissions of CO_2_ to the atmosphere; a variety of options would be available. A renewal period could be set at decadal or longer intervals. Conditions that define intentional and unavoidable (e.g., force majeure) forest carbon reversals and remedies for the parties are also agreed upon. No-fee registration of the project forest carbon products is included in the management plan, providing a transparent accounting, based on generally accepted accounting principles (GAAP). Actual agreements and contracts may vary according to each project.
